# Annual acknowledgement of manuscript reviewers

**DOI:** 10.1186/1471-2091-14-7

**Published:** 2013-03-21

**Authors:** Emilie Aime

**Affiliations:** 1 BioMed Central, Floor 6, 236 Gray’s Inn Road, London, WC1X 8HB, UK

## Abstract

**Contributing reviewers:**

The editors of *BMC Biochemistry* would like to thank all of our reviewers who have contributed to the journal in volume 13 (2012).

## 

Benedict Albensi

Canada

Brian Ayre

USA

Stephen Badylak

USA

Jesãºs Balsinde

Spain

Donatella Barra

Italy

Jürgen Bereiter-Hahn

Germany

Mikael Bjornstedt

Sweden

Stephen Bornemann

UK

David Braun

USA

Floyd Bryant

USA

Claudia Buitrago

Argentina

Michael Butcher

USA

Giampiero Cai

Italy

Claire Chaussade

UK

Kenneth Chavin

USA

Keping Chen

China

Heung-Chin Cheng

Australia

Pao-Yun Cheng

Taiwan

Anthony Clarke

Canada

Olivier Coux

France

Michael Czubryt

Canada

Elisabeth Daub

Canada

Claudio De Virgilio

Switzerland

Jeremy Derrick

UK

Arunkumar Dhayalan

India

Frank Dietz

Germany

Alexander Dikiy

Norway

Vernon Dolinsky

Canada

Wolfgang Dubiel

Germany

Weiyi Fang

China

Janet Finer-Moore

USA

Wolfgang Fischle

Germany

Peter Friedhoff

Germany

Lorinda Frylinck

South Africa

Jean-Marc Gallo

UK

Miguel Gijon

USA

Gary Goldberg

USA

Frank Grosse

Germany

Vincent Hascall

USA

Mark Helm

Germany

Emilio Hirsch

Italy

Kwang Yeon Hwang

South Korea

Yukio Ishimi

Japan

Yu Jau-Song

Taiwan

Juan Luis Jurat-Fuentes

USA

Le Kang

China

Tiebang Kang

China

Stephan König

Germany

Diane Krause

USA

Srikanth Kudithipudi

Germany

Thimo Kurz

UK

Ruiting Lan

Australia

Arnon Lavie

USA

Tsung-Ming Lee

Taiwan

Seng-Kee Leong

Australia

Wei Liu

USA

Jack Losso

USA

Stefan Lutz

USA

Tom Madej

USA

Hisao Masai

Japan

Julie Menetrey

France

Tony Miller

UK

Sang Hyun Min

South Korea

Taeko Miyagi

Japan

Hugo Monteiro

Brazil

Eugenio Monti

Italy

Stuart Edward Hanson Moore

France

James Morrissey

USA

Iouri Motorine

France

Voster Muchenje

South Africa

Martina Mühlenhoff

Germany

David O’Callaghan

France

Michael Overduin

UK

Alberto Passi

Italy

Mulchand Patel

USA

Lirong Peng

USA

Isabelle Petropoulos

France

Elah Pick

Israel

Lionel Pintard

France

David Plas

USA

Tassula Proikas-Cezanne

Germany

Qingsheng Qi

China

Chao-Nan Qian

China

Frederick Racke

USA

Philipp Rathert

Austria

Jaanus Remme

Estonia

Reza Saidi

USA

Suzana Salcedo

France

Kumar Sambamurti

USA

Honoo Satake

Japan

Supriya Sen

USA

Dejan Skorjanc

Slovenia

Mario Soberón

Mexico

Alexander Steinbuchel

Germany

Scot Stone

Canada

Pinghua Sun

China

Tadashi Suzuki

Japan

Pierre-Olivier Vidalain

France

Steve Winder

UK

Dimitris Xirodimas

France

Pengyuan Yang

China

Yukiko Yoshida

Japan

Jane Yu

USA

Chunping Zhao

USA

Ping Zhao

China

Boxiong Zhong

China

Lei Zhou

USA

